# Cardiovascular Damage in COVID-19: What We Know Two Years Later

**DOI:** 10.1007/s11886-022-01730-4

**Published:** 2022-06-25

**Authors:** Vincenzo Nuzzi, Eva Del Mestre, Alessia Degrassi, Daniel I. Bromage, Paolo Manca, Susan Piper, Jessica Artico, Piero Gentile, Paul A. Scott, Mario Chiatto, Marco Merlo, Nilesh Pareek, Mauro Giacca, Gianfranco Sinagra, Theresa A. McDonagh, Antonio Cannata

**Affiliations:** 1 Cardiothoracovascular Department, Azienda Sanitaria Universitaria Integrata Giuliano Isontina (ASUGI), University of Trieste, Trieste, Italy; 2 Department of Emergency Medicine, Azienda Sanitaria Universitaria Integrata Giuliano Isontina (ASUGI), Friuli-Venezia Giulia, Trieste, Italy; 3 Department of Cardiovascular Science, Faculty of Life Science and Medicine, King’s College London, London, UK; 4 Institute of Cardiovascular Science, Barts Heart Centre, Barts Health NHS Trust, University College London, West Smithfield, London, UK; 5 De Gasperis Cardio Center, Niguarda Hospital, ASST Grande Ospedale Metropolitano Niguarda, Milan, Italy; 6 UOC di Cardiologia UTIC, Ospedale Civile dell’Annunziata, Cosenza, Italy

**Keywords:** Cardiovascular injury, COVID-19, Acute cardiac damage, Biomarkers

## Abstract

**Purpose of the Review:**

The Coronavirus disease 2019 (COVID-19) pandemic has profoundly influenced cardiological clinical and basic research in the past two years. In the present review, we summarize the current knowledge on myocardial involvement in COVID-19, providing an overview on the incidence, the pathogenetic mechanisms, and the clinical implications of cardiac injury in this setting.

**Recent Findings:**

The possibility of heart involvement in patients with COVID-19 has received great attention since the beginning of the pandemic. After more than two years, several steps have been taken in understanding the mechanisms and the incidence of cardiac injury during COVID-19 infection. Similarly, studies globally have clarified the implications of co-existing heart disease and COVID-19.

**Summary:**

Severe COVID-19 infection may be complicated by myocardial injury. To date, a direct damage from the virus has not been demonstrated. The presence of myocardial injury should be systematically assessed for a prognostication purpose and for possible therapeutic implications.

## Introduction

The global pandemic caused by Coronavirus disease 2019 (COVID-19) is still a significant cause of morbidity and mortality, with a rapidly increasing number of infections and deaths worldwide [1]. Despite improvements in containing the spread of the virus, achieved by a massive campaign of vaccination, and in improving outcome of these patients, mainly using immunomodulatory drugs, several aspects of the Severe Acute Respiratory Syndrome Coronavirus 2 (SARS-CoV-2) are still unclear. In particular, cardiac involvement has been widely investigated in this setting, but findings have been inconclusive.

**Fig. 1:**
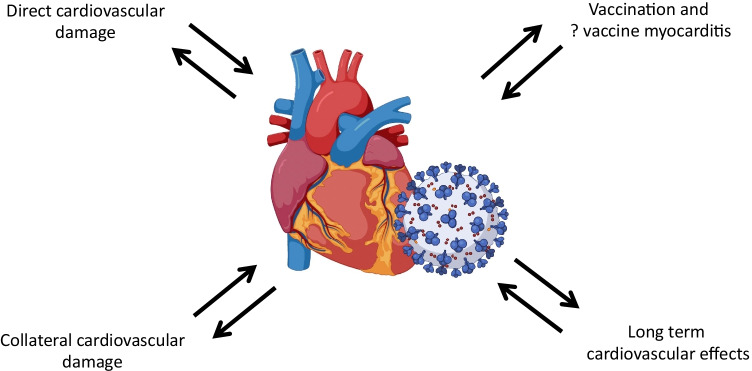
Summary of the cardiovascular effects of COVID

Although COVID-19 is mainly a respiratory syndrome, it may affect multiple organs including the heart. The myocardium is a sensitive tissue that may be damaged both by specific interaction with COVID-19 and general illness. Multiple and different mechanisms have been advocated to explain the possible primary involvement of cardiomyocytes [2]. In this review, we provide an overview of cardiac involvement during COVID-19, its clinical relevance, and the collateral damage of the pandemic on the cardiologic healthcare system (Fig. 1).

## Mechanisms of Cardiac Damage From SARS-CoV-2

The key mechanism that allows the virus to penetrate human cells is the bond between the spike protein, expressed on the viral coat, and the human receptor Angiotensin-Converting Enzyme 2 (ACE 2). ACE 2 is expressed, amongst others, on the surface of heart and blood vessels cells [3]. COVID-19, due to the mechanism needed to enter the host cells, may cause a downregulation of ACE 2, with a consequent inhibition of its physiological cardioprotective action. This process may therefore result in cardiovascular damage.

In addition, multiple indirect mechanisms of myocardial injury have been described [2]. Hypoxemia, caused by pneumonia and respiratory failure, is responsible for reactive oxygen species generation and subsequent oxidative stress and cell death, which may affect cardiac cells. Another fundamental mechanism concerns the systemic hyperinflammatory response, supported by a massive release of pro-inflammatory cytokines, called a cytokine storm. Moreover, infection may contribute to a pro-coagulative state that, together with an endothelial dysfunction and inflammation, can cause arterial and venous thromboembolism. Furthermore, the increased adrenergic activity related to the infection has positive inotropic and chronotropic effects, which may worsen the mismatch between oxygen supply and demand, resulting in relative myocardial ischemia [4].

Finally, a potential direct cardiotoxic effect of the SARS-CoV-2 virus is the occurrence of myocarditis. Myocarditis during COVID-19 has been variably described in case reports and case series, often without confirmatory diagnostic tests. Several post-mortem studies support the hypothesis that myocarditis is not a common histological finding in patients deceased for COVID-19 and may indicate that myocarditis is not an important feature of COVID-19 [5, 6]. One of the most representative cases of myocarditis described a patient presenting with cardiogenic shock, where endomyocardial biopsy was carried and it was proven myocardial localization of the coronavirus, but necrosis and virus particles inside the myocytes were not noted [7•]. To date, no studies have demonstrated the presence of viral genome in cardiomyocytes. Thus, the existence of COVID-myocarditis is debated and cannot be proven.

## Identifying Cardiovascular Damage From SARS-CoV-2

Several reports estimated the incidence of myocardial involvement in COVID-19 worldwide, but the results are quite contrasting [2, 4, 8••, 9–11]. This discrepancy may be related to different definitions adopted to define myocardial damage. Indeed, myocardial involvement during COVID-19 is a recent concept; therefore, several definitions have been considered.

Cardiac troponin is the main biomarker used to define acute myocardial injury [12]. Use of troponin has been translated from other settings that evaluate cardiac damage during extra-cardiac diseases, such as sepsis, bacterial pneumonia, and cerebrovascular events. Levels of high-sensitivity troponin above the reference limit were observed in a range from 7 to 40% of critically ill COVID-19 patients [8••]. This wide range of incidence may relate to different disease severities. Moreover, the presence of comorbidities may also contribute to the risk of myocardial injury during infection [8••, 9–11]. Another possible way to define cardiovascular damage is with natriuretic peptides. Natriuretic peptides are released in any condition that results in acute or chronic myocardial stress. For example, in a sub-analysis of the Italian Cardio-COVID registry, it was demonstrated that more than the half of patients with severe COVID-19 showed a natriuretic peptide level above the limit for detection of acute heart failure [13]. Whether natriuretic peptide releases correlates with heart failure in this setting is not yet known.

Finally, some evidence suggests that myocardial damage in COVID-19 may be identified by electrocardiogram and echocardiographic findings. Giustino et al. reported the prevalence of right ventricular systolic dysfunction (26%), left ventricular systolic dysfunction (18%), grade II and III diastolic dysfunction (13%), and pericardial effusion (7%) in two-thirds of patients [14]. More sophisticated echocardiographic techniques to identify subtle myocardial impairment include global longitudinal strain (GLS). In a Chinese cohort, decreased right ventricular GLS was strongly associated with increased morbidity and mortality [15].

## Outcomes of Patients With COVID-19 and Cardiovascular Involvement

The presence of cardiovascular damage in COVID-19 has been associated with unfavourable outcomes. The reasons are not fully understood but may include more severe COVID-19 disease with a higher degree of pneumonia damage, inflammation, pre-existing heart condition affecting prognosis, ICU care, and primary heart and vascular conditions during hospitalization. The occurrence of acute cardiac syndromes was proven to severely affect prognosis of COVID-19 patients. Indeed, pulmonary embolism, atrial arrhythmias, and acute heart failure were found to be strong prognosticators in this setting [16, 17]. In addition to acute cardiac complications of COVID-19, longer-term clinical sequelae on the heart after hospitalization for COVID-19 may contribute to worse outcomes, but there is little available evidence in this area.

Several studies have demonstrated that an elevated serum level of cardiac troponin is associated with worse outcomes, regardless of age, gender, oxygen saturation, heart failure history, and other potential confounders [8••, 9–11]. Interestingly, it was shown that myocardial injury may occur in patients without signs of myocardial involvement at admission [18]. The risk of in-hospital adverse outcomes amongst patients with cardiac involvement (defined by increased troponin levels only on day 2) is comparable to those with myocardial damage at admission. Similarly, patients at increased risk of in-hospital death can be identified by increased levels of natriuretic peptides, and the prognostic power of this biomarker was even higher if combined with troponin levels. Interestingly, NTproBNP or BNP values may be high not only in patients with acute or chronic heart failure, but also in patients without signs or symptoms of fluid overload [13].

COVID-19 patients with cardiovascular damage experience a high incidence of complications and require mechanical ventilation more frequently, leading to a longer hospitalisation, with the need of ICU stay or sub-intensive treatment [8••, 10, 16].

In an autopsy cohort of 40 patients deceased from COVID-19, all patients had chronic cardiac disease, but only two of them showed evidence of myocarditis and only one was affected by overt lymphocytic myocarditis. Interestingly, viral genome and Spike protein were not found in heart samples of patients with myocarditis. Authors suggested, supported by histopathological findings, that acute hypoxic cardiomyopathy, or the combination of acute hypoxia and pre-existing cardiac disease, along with a cytokine storm, is the main culprits of COVID-19-related cardiac injury [19].

Finally, the incidence of myocardial injury may be higher in patients with chronic cardiac conditions such as heart failure or atrial fibrillation (AF). These comorbidities are independent prognostic factors even after adjustment for elevated troponin levels [20, 21]. Concerning AF, new-onset AF during COVID-19 infection represents a biomarker of disease severity and is associated with poor outcome, similar to other settings [22].

## Collateral Cardiovascular Damage of the COVID-19 Pandemic

Not only has COVID-19 had putative direct effects on the heart, but the impact of the pandemic on healthcare systems has disrupted clinical care for several conditions, including CV disease [23, 24••]. The reconfiguration of care led on one side to the reduction in hospitalizations but increased in-hospital mortality for CV disorders and, on the other, to a higher incidence of out-of-hospital life threatening conditions [25]. During the pandemic, the reduction in hospitalisations for decompensated heart failure (HF) was associated with more severe signs and symptoms in HF patients, which was paralleled by worse in-hospital outcomes [26]. Similar results have been shown in different countries with varying degrees of impact from the pandemic [27]. A potential explanation of worse outcomes may be the relative reduction in hospital admission. In a recent meta-analysis, a decline in > 50% of hospitalisations compared to the previous year, was a predictor of adverse outcomes, independent of COVID-19 infection [27]. This suggests the reconfiguration of healthcare services and the disruption of care may have had a significant impact on patients with CV conditions.

Follow-up of patients discharged from hospital may have been similarly impacted. Patients admitted during the pandemic had worse long-term outcomes compared to those admitted the previous year [27]. This may reflect impediments to follow-up in primary and secondary care. Interestingly, the results observed during the first wave of the pandemic may be similar in other waves [28, 29].

## Assessment of Cardiovascular Damage: How and When

In patients admitted for respiratory failure COVID-related, myocardial injury does not necessarily indicate acute cardiac disease, such as acute coronary syndrome or heart failure, but is on the spectrum of a severe systemic condition [30–32]. Troponin measurement is a tool that allows an easy assessment of myocardial injury, and it is widely available and low cost and may provide important prognostic and clinical information in these patients.

A smaller number of studies focused on more specific definitions of myocardial damage, such as natriuretic peptides, echocardiography, strain analysis, and cardiac magnetic resonance. Except for cardiac magnetic resonance, whose prognostic associations in acute COVID-19 patients are unknown, all these different criteria defining myocardial damage had a strong association with outcome [8••, 15, 33, 34]. The results support the hypothesis that myocardial damage should be assessed in each patient with a risk stratification aim. However, in clinical practice in pandemic times it is unlikely that all COVID-19 unit patients may undergo a comprehensive cardiac evaluation (i.e. troponin and natriuretic peptides measurement, echocardiography, strain analysis, cardiac MR, etc.). Therefore, in patients without signs or symptoms of acute cardiac disease, it is reasonable to test at least biomarkers and to perform an ECG, as they are inexpensive and widely available tests. However, the presence of any abnormalities on these screening tests should not routinely prompt further testing, as evidences supporting this diagnostic flow chart. Indeed, the presence of a mild troponin release or signs of long-standing ECG abnormalities do not necessarily suggest an acute cardiovascular manifestation. On the other hand, echocardiography may be indicated in selected patients showing a predominant myocardial involvement (i.e. HF, arrhythmias, ACS, very high biomarkers increase, etc.), as they may implicate a therapeutic intervention [35]. In this scenario, acute myocarditis may be responsible for the myocardial damage. This issue has been reported in several case reports, some of them also testing myocardial tissue by endomyocardial biopsy or post-mortem autopsy [7•, 19]. However, in none of these cases a direct cause–effect relation between COVID-19 and acute cardiac inflammatory disease has been reported. Indeed, the presence of coronaviruses in the heart tissue was not related with a direct cellular damage from the virus. Therefore, recommendations for endomyocardial biopsy should be in accordance with the indications provided in previous consensus [36].

## Temporal Link Between COVID-19 Vaccination and Cardiovascular Damage

The presence of myocardial damage following COVID-19 vaccination has been extensively investigated. Several case reports of myocarditis and/or pericarditis following COVID-19 vaccination are available, even though the only link proven was a temporal connection [37, 38]. The estimated incidence of myocarditis in a large Israelian cohort is 2.13 cases per 100,000 subjects [39]. Interestingly, another study demonstrated that the incidence of myocarditis in a cohort of subjects undergoing COVID-19 vaccination is similar to the incidence in a matched cohort not exposed to COVID-19 vaccine. This finding underlines that the cardiac risk of COVID-19 vaccine is trivial, and the benefit predicted from vaccination outweighs the cardiac risk of COVID-19. Currently, a clinical trial is recruiting patients who experience heart involvement, defined as clinical signs or symptoms suggestive of myocarditis or myocardial injury within one month of COVID vaccine administration, after COVID-19 vaccination. The aim of the investigators is to define if these patients have a higher rate of myocardial inflammation on CMR or PET scan compared to those undergoing vaccination without signs or symptoms of myocardial injury [40].

Finally, myocardial damage has also been investigated in patients who have recovered from COVID-19. Fortunately, incident severe cardiac disease has not been reported after COVID-19 infection. However, advanced imaging techniques showed that a non-negligible share of patients recovered from COVID-19 have persistent myocardial involvement, identified by subtle abnormalities at advanced echocardiography or cardiac magnetic resonance several months after infection. Specifically, up to 58% of recovered patients had abnormal findings on CMR [41]. However, this is an association and causality cannot be inferred. Moreover, less expensive and more widely available methods, such as echocardiography deformation imaging, showed that up to 40% of recovered COVID-19 recovered patients had impaired RV or LV strain [42, 43]. It is important to note that the clinical implications of these findings are still unclear and therefore routine assessment of residual myocardial damage in asymptomatic individuals cannot yet be recommended. Further studies, such as more sophisticated imaging technique and prospective observational follow-up evaluations, are warranted to obtain information on possible cardiovascular outcomes incidence in these patients [44, 45].

## Clinical Management of Cardiovascular Damage

Cardiovascular damage has been extensively investigated in terms of its prognostic value, but, currently, no therapies have been tested specifically for patients with myocardial damage in COVID-19. Nor are clinical trials ongoing to explore possible therapies. Clinical trials in this setting are quite demanding as the emergency pandemic situation makes clinical research challenging. Moreover, the subset of patients with myocardial damage is limited and would require a routine assessment of this aspect in all patients admitted for COVID-19. Finally, the myocardial damage mechanisms have not been clearly defined yet, making it challenging to identify a specific drug for these patients. However, it may be speculated that if inflammation plays a key role, corticosteroids and, perhaps, anakinra may be considered in future clinical trials, acknowledging the possible common aetiology of pneumonia and heart damage. Indeed, despite in the RECOVERY trial information regarding acute cardiovascular injury of the study population lack, 27% of the patients had a history of cardiac disease [46]. Therefore, it is likely that corticosteroids are beneficial even in these patients. Anticoagulant therapy with direct oral anticoagulation was proven to be effective in reducing the mortality risk in patients with myocardial injury in patients after non-cardiac surgery, but the effect on the myocardial damage in COVID-19 is still unexplored [47]. Finally, ensuring evidence-based care of pre-existing and newly developed cardiovascular disease should be always pursued to reduce the risk of unfavourable cardiac outcomes. In Table 1 are reported the main implications of cardiovascular damage in COVID-19 patients.

**Table 1:** Types of cardiovascular damage of COVID and their management

**Type of cardiovascular damage**	**Clinical implications**	**Clinical management**
**Troponin elevation**	Higher risk of in-hospital complications and in-hospital death	Closer monitoring, higher intensity of care
**Natriuretic peptides elevation**	Higher risk of in-hospital complications and in-hospital death, possible acute heart failure	Monitoring of acute fluid retention and eventual targeted therapy
**LV systolic dysfunction**	Higher risk of in-hospital complications and in-hospital death, possible incidence of cardiac death	Closer monitoring, higher intensity of care, evaluation of the underlying etiology
**Pulmonary embolism**	Higher risk of in-hospital death	Specific therapy for pulmonary embolism, higher intensity of care
**Arrhythmias**	Higher risk of in-hospital death, possible incidence of thromboembolism	Specific therapy for cardiac arrhythmias
**Post-COVID subtle cardiac dysfunction**	Unknown	Needing for further research on these patients

At the moment, myocardial injury, taking into account its prognostic effect, may be used to stratify the risk of COVID-19 patients and detect those who may most benefit from admission in intensive care and other interventions. It is desirable that myocardial involvement would be included in multiparametric prognostic models aiming to predict the outcome of these patients.

Finally, post-COVID-19 myocardial injury is still a mostly unexplored field. Similarly, the presence of increased troponin levels after COVID infection deserves further research and to understand whether these findings are persistent even after a long period free from COVID-19 infection and whether these patients have a meaningful risk of clinical events.

## Lessons Learnt and Future Perspectives

In the future, the lessons learnt should be applied to different scenarios at different levels and have a potential high impact in managing further emergencies. The cardiovascular protection and investigation of cardiovascular damage and the public health messages are to preserve care of people with CV conditions.

Patients affected by cardiovascular conditions are extremely vulnerable and deserve dedicated services to prevent worse outcomes. Public health messaging should be targeted to find a balance between both limiting the spread of the virus and avoiding underestimation of potentially lethal conditions, such as cardiovascular deterioration.

Altogether, these changes might reduce the collateral damage of the COVID pandemic, enabling appropriate management of patients with cardiovascular conditions regardless of COVID. This pandemic is a substantial stress test for institutional resilience, innovation, and coordinated team working. Lessons learnt should now inform a more resilient approach to minimise cardiovascular collateral damage in future outbreaks but should also improve many other aspects of our routine work.
